# Comparison of Lower Genital Tract Microbiota in HIV-Infected and Uninfected Women from Rwanda and the US

**DOI:** 10.1371/journal.pone.0096844

**Published:** 2014-05-09

**Authors:** Lorie Benning, Elizabeth T. Golub, Kathryn Anastos, Audrey L. French, Mardge Cohen, Douglas Gilbert, Patrick Gillevet, Elisaphane Munyazesa, Alan L. Landay, Masoumeh Sikaroodi, Gregory T. Spear

**Affiliations:** 1 Department of Epidemiology, Johns Hopkins Bloomberg School of Public Health, Baltimore, Maryland, United States of America; 2 Departments of Medicine and Epidemiology & Population Health, Albert Einstein College of Medicine and Montefiore Medical Center, Bronx, New York, United States of America; 3 Ruth M. Rothstein CORE Center, Stroger Hospital of Cook County, Chicago, Illinois, United States of America; 4 Department of Medicine, Rush University Medical Center, Chicago, Illinois, United States of America; 5 Department of Immunology/Microbiology, Rush University Medical Center, Chicago, Illinois, United States of America; 6 Department of Environmental Science and Policy, George Mason University Microbiome Analysis Center, Manassas, Virginia, United States of America; 7 Department of Biomedical Services, Rwanda Biomedical Center National Reference Laboratory Division, Kigali, Rwanda; South Texas Veterans Health Care System and University Health Science Center San Antonio, United States of America

## Abstract

**Introduction:**

Previous studies have shown that alterations of the bacterial microbiota in the lower female genital tract influence susceptibility to HIV infection and shedding. We assessed geographic differences in types of genital microbiota between HIV-infected and uninfected women from Rwanda and the United States.

**Methods:**

Genera of lower genital tract bacterial microbiota were identified by high-throughput pyrosequencing of the 16S rRNA gene from 46 US women (36 HIV-infected, 10 HIV-uninfected) and 40 Rwandan women (18 HIV-infected, 22 HIV-uninfected) with similar proportions of low (0–3) Nugent scores. Species of *Lactobacillus* were identified by assembling sequences along with reference sequences into phylogenetic trees. Prevalence of genera and *Lactobacillus* species were compared using Fisher's exact tests.

**Results:**

Overall the seven most prevalent genera were *Lactobacillus* (74%), *Prevotella* (56%), *Gardnerella* (55%), *Atopobium* (42%), *Sneathia* (37%), Megasphaera (30%), and *Parvimonas* (26%), observed at similar prevalences comparing Rwandan to US women, except for *Megasphaera* (20% vs. 39%, p = 0.06). Additionally, Rwandan women had higher frequencies of *Mycoplasma* (23% vs. 7%, p = 0.06) and *Eggerthella* (13% vs. 0%, p = 0.02), and lower frequencies of *Lachnobacterium* (8% vs. 35%, p<0.01) and *Allisonella* (5% vs. 30%, p<0.01), compared with US women. The prevalence of *Mycoplasma* was highest (p<0.05) in HIV-infected Rwandan women (39%), compared to HIV-infected US women (6%), HIV-uninfected Rwandan (9%) and US (10%) women. The most prevalent lactobacillus species in both Rwandan and US women was *L. iners* (58% vs. 76%, p = 0.11), followed by *L. crispatus* (28% vs. 30%, p = 0.82), *L. jensenii* (20% vs. 24%, p = 0.80), *L. gasseri* (20% vs. 11%, p = 0.37) and *L. vaginalis* (20% vs. 7%, p = 0.10).

**Discussion:**

We found similar prevalence of most major bacterial genera and *Lactobacillus* species in Rwandan and US women. Further work will be needed to establish whether observed differences differentially impact lower genital tract health or susceptibility to genital infections.

## Introduction

Many reproductive-aged women have lower genital tract microbiota that can be classified as either "healthy" microbiota or bacterial vaginosis (BV) [1]–[6]. However, there is considerable heterogeneity within these classifications. For example, while healthy microbiota generally are comprised mostly of *Lactobacillus* spp., in different women *L. crispatus, L. iners*, *L. jensenii* or *L. gasseri* can be the dominant species [7]–[9]and there are significant differences in the beneficial effects of each [10]–[16]. BV can also vary dramatically between women. For example, Fredricks et al. showed, by sequencing 16S ribosomal RNA genes, that while *Gardnerella vaginalis* was a dominant bacterium in many cases of BV (comprising as much as 49% of the bacterial sequences), in some cases *G. vaginalis* was almost absent (1–2% of sequences) [8]. Similarly, while most women with BV had microbiota with substantial proportions of *Prevotella* sequences (25–30% of sequences), some had no detectable *Prevotella* sequences. A substantial heterogeneity in BV has been confirmed in other recent studies using molecular-based methods [7], [17].

Cross-sectional and longitudinal epidemiologic studies show that BV is associated with increased susceptibility to infection with HIV [18], [19] and other sexually transmitted diseases (STD) [20], [21]. BV is also associated with increased genital shedding of HIV in women [22]–[25]. While BV is associated with these infections, an unresolved question is whether heterogeneity within BV is associated with HIV.

The 2010 UNAIDS Global Report estimated that in 2009 the prevalence of HIV in Rwandans aged 15–49 was 2.9% (2.5%–3.3%), five times higher than the estimated rate of 0.6% (0.4%–0.8%) in the United States (US) [26]. The rate of 1.9% (1.3%–2.3%) among young Rwandan women (15–24) was 10 times higher than the rate of 0.2% (0.1%–0.3%) among young US women [26]. Differences in types of genital microbiota are potentially among a number of factors that contribute to these large disparities in HIV prevalence between Rwanda and the US. The purpose of the current pilot study was to investigate the heterogeneity of genital bacterial microbiota between HIV-infected and –uninfected Rwandan and US women. Organisms found to be more prevalent in one country, particularly among HIV-infected women compared to HIV-uninfected women, may contribute to HIV susceptibility or shedding. Associations between these organisms and HIV viral load in the female genital tract could be targeted in larger future studies. Although some genital bacteria are easily cultured (e.g. *Gardnerella*), it has become clear recently that many genital bacteria are either unculturable (e.g. *Sneathia*) or are difficult to culture since they are anaerobic (e.g. *Mobiluncus* and *Atopobium*). Therefore, we used high-throughput pyrosequencing of the 16S rRNA gene to identify the genital microbiota of women in this study. Previous 16S-based high-throughput sequencing studies of microbiota have been performed on US [7], [27], [28], Chinese [29], and Tanzanian women [17]. We directly compared the genital microbiota between US and Rwandan women, overall and stratified by HIV serostatus.

## Materials and Methods

### Study Population

The study population of 86 women came from two parent studies, the Women's Interagency HIV Study (WIHS) and the Rwandan Women's Interassociation Study and Assessment (RWISA). Both the WIHS and the RWISA are observational cohort studies of HIV-infected and uninfected women. Semiannual study visits included extensive interviews, specimen collection, and clinical examinations. The WIHS enrolled a total of 3766 women (2791 HIV-infected, 975 HIV-uninfected) in two waves, from 10/1994 to 11/1995 [30] and 10/2001 to 9/2002 [31], for the purpose of investigating the progression of HIV infection and associated diseases, such as diabetes and cardiovascular disease, among HIV-infected and demographically similar HIV-uninfected women in the US. The RWISA enrolled 936 women (710 HIV-infected, 226 HIV-uninfected) in 2005 for the purpose of investigating responses to antiretroviral therapy in Rwandan women, including potential impact of their experiences during the 1994 Rwandan genocide [32]. The 46 WIHS participants (36 HIV-infected, 10 HIV-uninfected) included in the current study have been described in two previously conducted substudies [9], [28]. The 40 RWISA participants were selected for the current study to have Nugent scores similar to the WIHS participants. All data on sexual behavior and STD history were missing for one Rwandan participant.

### Ethics Statement

The study was conducted in accordance with protocols approved by the Rwandan National Ethics Committee and the Institutional Review Board (IRB) of Montefiore Medical Center, Bronx, NY, USA for RWISA participants and guidelines of the US Department of Health and Human Services and the IRBs of each of the WIHS institutions and the WIHS Executive Committee for WIHS participants. Written informed consent was obtained for all participants from both cohorts.

### Laboratory methods

Genital tract samples for both studies were collected by cervicovaginal lavage (CVL) performed by irrigation of the cervix with 10 mL of nonbacteriostatic sterile saline, followed by aspiration from the posterior fornix. All CVL samples were gently vortexed to evenly distribute cells before they were aliquotted; RWISA CVL samples were processed within 2 hours of collection and WIHS CVL samples were held on ice until processing within 6 hours of collection. CVL samples from both studies were stored at −70°C. Specimens from HIV-infected WIHS participants were collected between 1/1995 and 4/1997, from HIV-uninfected WIHS participants between 10/2005 and 3/2006, and from all RWISA participants between 5/2005 and 11/2005. The methods used for DNA isolation and multitag pyrosequencing have been described elsewhere [33], [34]. Briefly, bar-coded primer sets each containing the 27F and 355R 16S rRNA gene primers were used. Only forward reads were used to identify bacteria using the Bayesian Classifier provided by the Ribosomal Database II Project (RDP 10). For each subject, analysis was performed on bacterial taxa found at ≥1% of the total sequences with the premise that the most abundant taxa contribute most significantly to the functionality of the microbial community. The average number of sequences obtained per sample was 3857 (range 895–7866) for HIV-uninfected Rwandan women, 3563 (range 491–7016) for HIV-infected Rwandan women, 1412 (range 224–2707) for HIV-uninfected US women, and 579 (249–1683) for HIV-infected US women. Species of *Lactobacillus* were identified by assembling sequences along with reference sequences into phylogenetic trees with a 96% overlap identity and 80% confidence threshold using Geneious Pro 4.6.1 software (Auckland, New Zealand). Reference sequences used included AF257097 (*L. crispatus*), M58820 (*L. gasseri*), AF243176 (*L. jensenii*), AF243177 (*L. vaginalis*), and AY526083 (*L. iners*).

### Statistical methods

**Figure 1:**
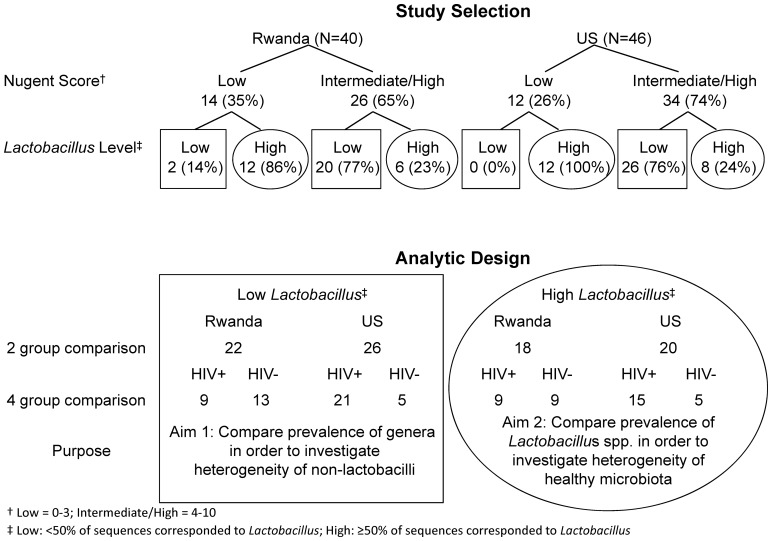
Study selection and analytic design.

Figure 1 shows the study selection and analytic design. Rwandan and US women were selected and frequency matched on the basis of Nugent score. Women were subsequently stratified by *Lactobacillus*-predominance versus non-*Lactobacillus* predominance.

There were two analytic aims, to investigate the heterogeneity of microbiota associated with BV (Aim 1) and to investigate the heterogeneity of healthy microbiota (Aim 2). For each aim we conducted two separate comparisons. First we compared all Rwandan and US women. Secondly, we compared by country and HIV status (four groups): HIV-infected Rwandan (RW+), HIV-uninfected Rwandan (RW-), HIV-infected US (US+), and HIV-uninfected US (US-) women. We compared prevalence, defined as the proportion of women in whom a given genus or species was present. A genus or species was considered present in a woman if at least 1% of all her sequences corresponded to it.

Fisher's exact tests were used to determine differences in the prevalence of individual genera and *Lactobacillus* spp. and to compare categorical demographic and behavioral characteristics. Mann-Whitney *U* tests were used to compare continuous demographic and behavioral characteristics. *P* values less than 0.05 were considered to be statistically significant and *P* values between 0.05 and 0.10, marginally significant. All analyses were conducted in SAS version 9 (SAS, Cary, North Carolina, USA).

## Results

Table 1 shows a comparison of demographic and behavioral characteristics between Rwandan and US women, stratified by *Lactobacillus* level. In general, US women had more sexual experience and higher proportions of STDs than Rwandan women. Differences between countries tended to be larger and more statistically significant among women with low *Lactobacillus*, especially for genital warts, syphilis, and PID, than among women with high *Lactobacillus*. The higher prevalence of HIV infection among US women versus Rwandan women overall (78% vs. 45%, p = 0.002) was reflected in each stratum and, again, was more pronounced among women with low *Lactobacillus*. None of the 18 HIV-infected Rwandan women had used antiretroviral medication (ART) compared to 19 (53%) of the 36 HIV-infected US women who were naïve to ART. Specimens from Rwandan women were collected before wide ART availability in Rwanda and between January 1995 and April 1997 for US women, when ART use was not yet optimal. CD4^+^ cell count was similar between RW+ and US+ women (218 vs. 254, p = 0.54). Log_10_ HIV RNA viral load was also similar (4.77 vs. 4.61, p = 0.76).

**Table 1:** Comparison of demographic and behavioral characteristics.

	Women with low *Lactobacillus* †			Women with high *Lactobacillus* †		
	Rwanda (N = 22)	US (N = 26)		Rwanda (N = 18)	US (N = 20)	
Characteristic	N (%) or Med (IQR)	N (%) or Med (IQR)	P-value*	N (%) or Med (IQR)	N (%) or Med (IQR)	P-value*
*HIV-seropositive*	9 (41)	21 (81)	0.01	9 (50)	15 (75)	0.18
*Age (years)*	40 (35–51)	38 (33–41)	0.22	37 (35–46)	36.5 (33.5–43)	0.57
*Marital status*						
Married or living with partner	7 (32)	10 (43)	0.001	6 (33)	6 (32)	0.01
Widowed	13 (59)	2 (9)		10 (56)	2 (11)	
Divorced/Annulled/Separated	2 (9)	7 (30)		1 (6)	5 (26)	
Never married	0	4 (17)		1 (6)	6 (32)	
**Sexual behavior**						
*Age at 1st sex (years)*	19 (18–21)	15 (13–17)	0.0003	18 (17–21)	15 (13–17)	0.01
*Lifetime male sex partners*	2 (1–3)	10.5 (5–50)	<0.0001	2 (1–5)	10 (6–37.5)	0.001
*Male sex partners past 5 years*	1 (0–1)	3 (1–10)	<0.0001	1 (0–1)	3 (1–4)	0.0004
*Male sex partners past 6 months*	0.5 (0–1)	1 (1–1)	0.04	0 (0–1)	1 (0–1)	0.21
*Condom use in past 6 months*						
No male partners	11 (50)	4 (16)	0.005	9 (53)	7 (39)	0.20
≥1 male partner, always	2 (9)	13 (52)		1 (6)	5 (28)	
≥1 male partner, sometimes	2 (9)	3 (12)		2 (12)	4 (22)	
≥1 male partner, never	7 (32)	5 (20)		5 (29)	2 (11)	
*Contractual sex ever*	7 (32)	14 (54)	0.15	1 (6)	7 (35)	0.05
*Contractual sex past 6 months*	3 (15)	0	0.08	0	1 (6)	1.00
**History of STD** ‡						
*Trichomonas*	12 (57)	10 (38)	0.25	9 (56)	9 (47)	0.74
*Gonorrhea*	5 (23)	7 (27)	1.00	1 (6)	5 (26)	0.18
*Herpes*	2 (9)	6 (23)	0.26	0	2 (10)	0.49
*Genital warts*	0	7 (27)	0.01	1 (6)	4 (21)	0.34
*Syphilis*	1 (5)	9 (35)	0.01	0	1 (5)	1.00
*Chlamydia*	0	3 (12)	0.24	1 (6)	4 (20)	0.35
*PID*	0	6 (23)	0.02	0	2 (11)	0.49
*Any STD*	14 (64)	23 (88)	0.08	9 (56)	17 (85)	0.07
*Multiple STDs*	8 (38)	16 (62)	0.15	5 (31)	10 (50)	0.32

Note: %, column percent; Med, median; IQR, interquartile range; Contractual sex, exchanged sex for money, drugs, or shelter; STD, sexually transmitted disease; PID, pelvic inflammatory disease.

† Low: 0–49% of sequences correspond to *Lactobacillus*; high: ≥50% of sequences correspond to *Lactobacillus*.

‡ Reported by participant at baseline visit.

*Reported *P* values were obtained from Fisher's exact tests for categorical variables and from Mann-Whitney *U* tests for co.

### Types of genital microbiota are similar between Rwandan and US women, overall

To compare the types of genital microbiota found in Rwandan and US women, genital tract samples obtained from each of the 86 women were used to identify bacterial genera by pyrosequencing of the 16S rRNA gene. Of the 86 prevalent genera identified across all 86 women (Table S1), 17 were unique to Rwandan women, 33 were unique to US women, and 36 were common to both groups. These 86 genera accounted for a median of 97% of all sequences (range 90–100%) among Rwandan women and 96% (90–100%) among US women (p = 0.12). Among Rwandan women, the median number of genera was 4.5 (range 1–15) compared to a median of 7.5 (range 1–23) for US women (p = 0.33).

**Figure 2:**
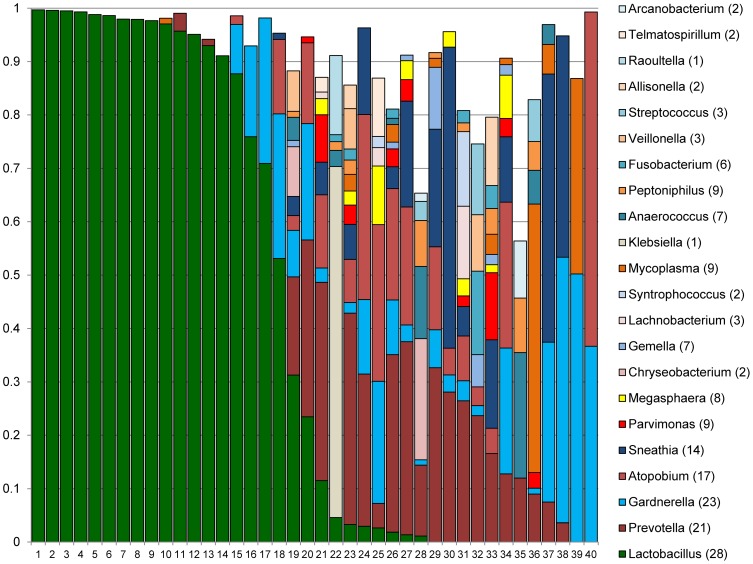
Distributions of major genera in Rwandan women. Stacked columns for each of the 40 individual women show the distributions of the proportion of sequences corresponding to a given genus. All 22 genera with proportions of at least 0.1 in at least one woman were included. Numbers in parentheses in the legend indicate the number of women in whom the genus was prevalent.

**Figure 3:**
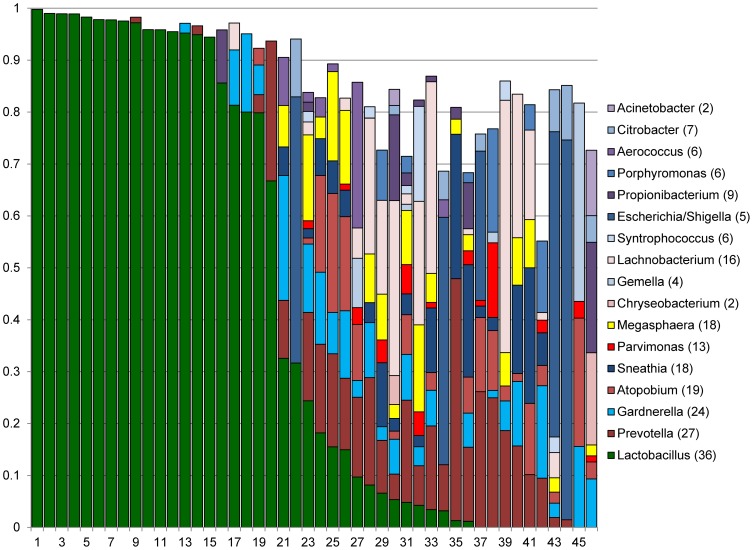
Distributions of major genera in US women. Stacked columns for each of the 46 individual women show the distributions of the proportion of sequences corresponding to a given genus. All 17 genera with proportions of at least 0.1 in at least one woman were included. Numbers in parentheses in the legend indicate the number of women in whom the genus was prevalent.

Figures 2 and 3 show the distributions of the most dominant genera in Rwandan and US women, respectively. Eleven genera comprised at least 10% of sequences in at least one Rwandan and at least one US woman: *Lactobacillus, Prevotella, Gardnerella, Atopobium, Sneathia, Parvimonas, Megasphaera, Chryseobacterium, Gemella, Lachnobacterium*, and *Syntrophococcus* (Figures 2 and 3). Proportions of these genera did not differ between Rwandan and US women, except for *Lachnobacterium* and *Megasphaera* (data not shown).

Overall, the seven most prevalent genera were *Lactobacillus* (74%), *Prevotella* (56%), *Gardnerella* (55%), *Atopobium* (42%), *Sneathia* (37%), *Megasphaera* (30%), and *Parvimonas* (26%) (Table S1). Prevalences were similar between countries for all except *Megasphaera* (20% Rwanda vs. 39% US, p = 0.06). The most prevalent *Lactobacillus* species in both Rwandan and US women was *L. iners* (58% vs. 76%, p = 0.11), followed by *L. crispatus* (28% vs. 30%, p = 0.82), *L. jensenii* (20% vs. 24%, p = 0.80), *L. gasseri* (20% vs. 11%, p = 0.37) and *L. vaginalis* (20% vs. 7%, p = 0.10) (Table S2).

### Prevalence of some microbiota associated with BV higher among US women

Table 2 shows the 28 most prevalent genera (of 83) observed among the 48 women with low *Lactobacillus*. The table is grouped by high (≥40%), moderate (16–39%), and low (10–15%) overall prevalence, and further stratifies by geography and HIV status. Rwandan women had lower prevalence of 10 genera (*Megasphaera*, *Lachnobacterium*, *Allisonella*, *Mobiluncus*, *Propionibacterium*, *Citrobacter*, *Achromobacter*, *Escherichia/Shigella*, *Enterobacter*, and *Phocaeicola*) and higher prevalence of *Mycoplasma* and *Eggerthella*, compared to US women. Prevalences for the other 16 genera were not significantly different between the two countries. Results were similar for the four group comparison (RW+, RW−, US+, US−). Of genera observed in both countries, *Megasphaera*, *Lachnobacterium* and *Allisonella* were higher in US+ and US−; *Asaccharobacter* was higher in RW+ and US+; *Mycoplasma* was highest in RW+; *Mobiluncus* and *Dialister* were highest in US−; and *Propionibacterium* was highest in US+.

**Table 2:** Comparison of the most prevalent genera among women with genital microbiota having 0–49% of sequences corresponding to *Lactobacillus*.

					Rwanda		US		
	Overall	Rwanda	US		HIV+	HIV−	HIV+	HIV−	
	N = 48	N = 22	N = 26		N = 9	N = 13	N = 21	N = 5	
Genus	Prevalence†	Prevalence†		*P* value*	Prevalence†				*P* value*
**High**									
*Prevotella*	88	86	88		78	92	86	100	
*Gardnerella*	81	86	77		89	85	76	80	
*Atopobium*	69	68	69		67	69	71	60	
*Sneathia*	65	59	69		78	46	67	80	
*Lactobacillus*	54	45	62		33	54	67	40	
*Megasphaera*	54	36	69	0.041	33	38	62	100	0.059
*Parvimonas*	46	41	50		44	38	57	20	
*Peptoniphilus*	40	41	38		22	54	48	0	
**Moderate**									
*Lachnobacterium*	38	14	58	0.003	0	23	48	100	0.001
*Allisonella*	33	9	54	0.002	22	0	52	60	0.003
*Porphyromonas*	25	27	23		33	23	19	40	
*Asaccharobacter*	25	14	35		33	0	38	20	0.043
*Mycoplasma*	23	36	12	0.082	67	15	10	20	0.007
*Gemella*	23	32	15		33	31	19	0	
*Anaerococcus*	23	32	15		22	38	19	0	
*Mobiluncus*	23	9	35	0.045	11	8	24	80	0.014
*Dialister*	19	14	23		22	8	14	60	0.088
*Propionibacterium*	19	5	31	0.028	0	8	38	0	0.039
*Fusobacterium*	17	27	8		33	23	10	0	
*Proteiniphilum*	17	14	19		22	8	19	20	
*Aerococcus*	17	9	23		11	8	29	0	
*Syntrophococcus*	17	9	23		0	15	19	40	
**Low**									
*Citrobacter*	15	0	27	0.011	0	0	33	0	0.017
*Achromobacter*	13	0	23	0.025	0	0	29	0	0.048
*Eggerthella*	10	23	0	0.015	0	38	0	0	0.003
*Escherichia/Shigella*	10	0	19	0.054	0	0	24	0	
*Enterobacter*	10	0	19	0.054	0	0	24	0	
*Phocaeicola*	10	0	19	0.054	0	0	14	40	0.062

† Percentage of women with genital microbiota having ≥1% of sequences corresponding to each genus listed. All genera with prevalence ≥19% in at least one group were included.

*Reported *P* values were obtained from Fisher's exact tests (only *P* values <0.10 are shown).

### Prevalence of *L. crispatus* is low among HIV-infected women

Among the 38 women with high *Lactobacillus*, only 17 genera in addition to *Lactobacillus* were observed. Of those, only three were observed at prevalences higher than 10% (*Gardnerella* 21%, *Prevotella* 16%, and *Ureaplasma* 11%). Therefore, we restricted analysis to *Lactobacillus* spp. among these women (Table 3, top), contrasting them to the 26 women with 1–49% of sequences corresponding to *Lactobacillus* (Table 3, bottom). Both halves of Table 3 show that overall *L. iners* was the dominant *Lactobacillus* spp. The diversity of *Lactobacillus* spp. was similar among the 18 Rwandan women and 20 US women with high *Lactobacillus* and the 10 Rwandan women with low *Lactobacillus*. However, there was almost no diversity in *Lactobacillus* spp. among the 16 US women with low *Lactobacillus*. Taking HIV into account revealed significantly lower prevalence of *L. crispatus* among all HIV-infected women with low *Lactobacillus* (p = 0.03). Among women with high *Lactobacillus*, the prevalence of *L. crispatus* was lower among Rwandan HIV-infected women but not US HIV-infected women.

**Table 3:** Comparison of the prevalence of *Lactobacillus* spp. among women with genital microbiota having ≥1% of sequences corresponding to *Lactobacillus*.

	Women with ≥50% of sequences corresponding to *Lactobacillus*								
					Rwanda		US		
	Overall	Rwanda	US		HIV+	HIV−	HIV+	HIV−	
	N = 38	N = 18	N = 20		N = 9	N = 9	N = 15	N = 5	
Species	Prevalence‡	Prevalence‡		*P* value*	Prevalence‡				*P* value*
*L. iners*	87	78	95	0.17	78	78	93	100	0.53
*L. crispatus*	50	39	60	0.33	22	56	60	60	0.33
*L. jensenii*	39	28	50	0.20	22	33	47	60	0.48
*L. gasseri*	24	28	20	0.71	11	44	27	0	0.29
*L. vaginalis*	21	28	15	0.44	11	44	20	0	0.28
L. other	76	78	75	1.00	56	100	67	100	0.05

	Women with 1–49% of sequences corresponding to *Lactobacillus* †								
					Rwanda		US		
	Overall	Rwanda	US		HIV+	HIV−	HIV+	HIV−	
	N = 26	N = 10	N = 16		N = 3	N = 7	N = 14	N = 2	
Species	Prevalence‡	Prevalence‡		*P* value*	Prevalence‡				*P* value*
*L. iners*	96	90	100	0.38	67	100	100	100	0.19
*L. crispatus*	23	40	13	0.16	0	57	7	50	0.03
*L. jensenii*	15	30	6	0.26	33	29	7	0	0.35
*L. gasseri*	15	30	6	0.26	33	29	7	0	0.35
*L. vaginalis*	12	30	0	0.05	33	29	0	0	0.11
L. other	77	70	81	0.64	67	71	93	0	0.02

† 1–31% of sequences among Rwandan women compared to 1–33% of sequences among US women.

‡ Percentage of women with genital microbiota having ≥1% of sequences corresponding to each species listed.

*Reported *P* values were obtained from Fisher's exact tests.

## Discussion

This exploratory pilot study was the first direct comparison of the types of lower genital tract microbiota of US and African women using pyrosequencing of the 16S rRNA gene. The major finding was an overall similarity in the types and prevalences of genital microbiota between US and Rwandan women, despite differences in sexual behaviors and STD history between the groups. This finding is congruous with previous studies. A large epidemiological study of West African women with BV also showed their genital microbiota was similar to that of women in developed countries [35]. Another study comparing phenotypic characteristics of microbiota, including production of acid and H_2_O_2_, from East African commercial sex workers and Canadian adolescents also noted similarity, reinforcing what previous work had suggested regarding the most frequently observed vaginal microorganisms across all groups of women studied around the world [36]. This suggests the possibility that the heterogeneity of the most common microbiota associated with BV is not largely impacted by sexual history or geography.

Regarding genera with dissimilar prevalences among US and Rwandan women, the higher frequency of *Mycoplasma* among HIV-infected Rwandan women could have important implications for HIV transmission. For example, a prospective study of women in the East African countries of Zimbabwe and Uganda found that the risk of HIV acquisition was significantly higher in those with *M. genitalium* [19]. Likewise in Kenya, women with high *M. genitalium* organism burdens were more likely to have measurable cervical HIV-1 DNA than were *M. genitalium*-negative women [37].

The coliform bacilli *Escherichia* and closely related *Shigella*, *Enterobacter*, and *Citrobacter* are members of the normal intestinal flora of humans [38], [39]. A limited number of species, including *E. coli*, *Enterobacter aerogenes*, *Enterobacter cloacae*, *S. dysenteriae*, and *S. flexneri* are responsible for most of the infections which cause major health problems around the world. In the vagina, these bacteria are the primary cause of urinary tract infections, particularly *E. coli* [40], [41]. Interestingly, these genera were prevalent only in US HIV-infected women in our study. This finding is intriguing, especially given the differences in sexual histories and STD prevalence between US and Rwandan women. Vaginal colonization with coliforms could be a marker of recent sexual activity. Future studies could investigate the association between these coliforms and markers of recent sexual activity such as PSA, RSID, and Y chromosome PCR.

No studies have shown any human disease to be associated with *Lachnobacterium*, *Allisonella*, *Asaccharobacter*, or *Phocaeicola*. *Propionibacterium* spp. can cause skin infection [42]. *Achromobacter* spp. have been associated with cystic fibrosis and immunosuppression [43]. However, the clinical significance, if any, in the human female genital tract for these bacteria is unknown. Studies of the gut microbiome show that individuals can have highly divergent types of microbiota, although the functional profiles are similar [44]. It is possible that in the genital tract, some of the differences we observed in types of microbiota also have similar functions.

Colonization with lactobacilli, particularly the H_2_O_2_-producing species *L. crispatus* and *L. jensenii*, has a protective effect against the acquisition of BV [10]–[16]. Macklaim et al. showed that *L. iners* has the ability to survive in the vagina under varying conditions [13]. *L. iners* was the dominant species among all women in our study, followed by *L. crispatus* and *L. jensenii*. The lower prevalence of *L. crispatus* that we observed among all HIV-infected women from Rwanda, and among some HIV-infected women from the US, could be clinically important if validated in a larger study.

It should be noted that this study was exploratory and limited by small sample size. However, although our results may not be generalizable to all Rwandan and US women, our samples were obtained from two established parent studies (WIHS and RWISA), including both HIV-infected and uninfected women, both with sound protocol and infrastructure [30]–[32]. Except for *Lactobacillus* spp., our analysis was conducted at the level of genera. It is therefore possible that while we observed similarities between countries in the most common genera, there could be important differences between countries at the species level. We have identified several genital bacterial microbiota (*Mycoplasma*, *Escherichia*/*Shigella*, *Enterobacter*, *Citrobacter*) which might be targeted in future studies using genus or species-specific PCR to investigate the association between bacterial load and HIV shedding and markers of recent sexual activity. Despite these limitations, this study revealed surprising similarities in the microbiota of US and Rwandan women, as well as some interesting differences that warrant further exploration. Additionally, it would be informative to investigate the clinical significance of these organisms.

## Supporting Information

Table S1 **Prevalence of all 86 observed genera.**
(XLSX)

Table S2 **Prevalence of** ***Lactobacillus*** **spp.**
(XLSX)
